# Redox engineering by ectopic expression of glutamate dehydrogenase genes links NADPH availability and NADH oxidation with cold growth in *Saccharomyces cerevisiae*

**DOI:** 10.1186/s12934-015-0289-2

**Published:** 2015-07-09

**Authors:** Lidia Ballester-Tomás, Francisca Randez-Gil, Roberto Pérez-Torrado, Jose Antonio Prieto

**Affiliations:** Department of Biotechnology, Instituto de Agroquímica y Tecnología de los Alimentos (CSIC), Avda. Agustín Escardino 7, 46980 Paterna, Valencia Spain

**Keywords:** Wine yeast, Winemaking, Low temperature, Fermentation, Tryptophan, ROS accumulation, *GDH1*, *GDH2*, *GPD1*, *GRE3*

## Abstract

**Background:**

Cold stress reduces microbial growth and metabolism being relevant in industrial processes like wine making and brewing. Knowledge on the cold transcriptional response of *Saccharomyces cerevisiae* suggests the need of a proper redox balance. Nevertheless, there are no direct evidence of the links between NAD(P) levels and cold growth and how engineering of enzymatic reactions requiring NAD(P) may be used to modify the performance of industrial strains at low temperature.

**Results:**

Recombinant strains of *S. cerevisiae* modified for increased NADPH- and NADH-dependent Gdh1 and Gdh2 activity were tested for growth at low temperature. A high-copy number of the *GDH2*-encoded glutamate dehydrogenase gene stimulated growth at 15°C, while overexpression of *GDH1* had detrimental effects, a difference likely caused by cofactor preferences. Indeed, neither the Trp^−^ character of the tested strains, which could affect the synthesis of NAD(P), nor changes in oxidative stress susceptibility by overexpression of *GDH1* and *GDH2* account for the observed phenotypes. However, increased or reduced NADPH availability by knock-out or overexpression of *GRE3*, the NADPH-dependent aldose reductase gene, eliminated or exacerbated the cold-growth defect observed in YEpGDH1 cells. We also demonstrated that decreased capacity of glycerol production impairs growth at 15 but not at 30°C and that 15°C-grown baker’s yeast cells display higher fermentative capacity than those cultivated at 30°C. Thus, increasing NADH oxidation by overexpression of *GDH2* would help to avoid perturbations in the redox metabolism induced by a higher fermentative/oxidative balance at low temperature. Finally, it is shown that overexpression of *GDH2* increases notably the cold growth in the wine yeast strain QA23 in both standard growth medium and synthetic grape must.

**Conclusions:**

Redox constraints limit the growth of *S. cerevisiae* at temperatures below the optimal. An adequate supply of NAD(P) precursors as well as a proper level of reducing equivalents in the form of NADPH are required for cold growth. However, a major limitation is the increased need of oxidation of NADH to NAD^+^ at low temperature. In this scenario, our results identify the ammonium assimilation pathway as a target for the genetic improvement of cold growth in industrial strains.

**Electronic supplementary material:**

The online version of this article (doi:10.1186/s12934-015-0289-2) contains supplementary material, which is available to authorized users.

## Background

Variations in ambient temperature are a common phenomenon in nature that influences the microbial growth and metabolism. Temperature drops modifies the molecular topology, the enzyme kinetics, and increases the molecular order of membrane lipids [1, 2], affecting key cellular processes as transcription, translation and membrane-associated activities [3]. Cold is also relevant for the industrial exploitation of microorganisms. Processes involving yeasts, like brewing and some wine fermentations, take place at temperatures around 10–12°C, which is far below the optimal temperature of this organism (~28°C). Therefore, understanding the mechanisms of cold survival and adaptation is of great interest for both basic and applied aspects.

The essential coenzymes nicotinamide adenine dinucleotides, NAD and NADP, participate in key redox reactions and contribute to maintaining cell fitness and genome stability [4]. Factors regulating their metabolism and homeostasis become thus crucial in providing metabolic flexibility and determining a proper cellular response to environmental changes [5]. Indeed, NAD-related genes have been identified as factors governing yeast cold growth [6]. Consistent with this, tryptophan metabolism genes, which are involved in the novo biosynthesis of NAD and NADP [5, 7], have been traditionally linked to cold tolerance in *Saccharomyces cerevisiae* [3]. Thus, many cold-sensitive mutants are tryptophan auxotrophs, have mutations in tryptophan permeases or are affected in tryptophan biosynthesis [8]. Nevertheless, there is no experimental evidence that NAD and/or NADP levels are limiting for cold growth or that the maintenance of an optimal balancing of reduced and oxidized forms preserves and promotes a proper response to low temperatures.

The need of a dynamic regulation of NAD metabolism and homeostasis at low temperature might be linked to the control of increased reactive oxygen species (ROS). Previous studies have demonstrated that a downward shift in the growth temperature of *S. cerevisiae* from 30 to 10°C increases the intracellular levels of H_2_O_2_ [9] and induces an antioxidant response [10, 11], specifically of genes regulated by the transcription factor Yap1 [9]. Several genes involved in detoxifying ROS and defense against oxidative stress such as catalase (*CTT1*), glutaredoxin (*TTR1*), thioredoxin (*PRX1*) and glutathione transferase (*GTT2*), are also induced at extremely low (4°C) temperature [12]. Unregulated ROS could result from reduced oxygen availability and respiration rate associated with low temperatures, since improper mitochondrial function is the main source of oxidative stress [13]. Consistent with this, typical anaerobiosis marker genes, like those of the DAN/TIR gene family, *TIP1*, *TIR1*, *TIR2*, and *TIR4* are up-regulated in cold-shocked cells [14]. Moreover, cold exposure results in a higher degree of catabolite repression [15] and increased fermentative capacity [16]. The cold-induced respiratory dysfunction could also impair the oxidation of NADH mitochondrial affecting the intracellular compartmentalization of NAD pools [5] and contributing to the redox imbalance. Consistent with this idea, *ADH3*, which encodes an ethanol-acetaldehyde redox shuttle involved in the transfer of redox equivalents from the mitochondria to the cytosol [17], has been described as implied in cold tolerance [6].

**Figure 1:**
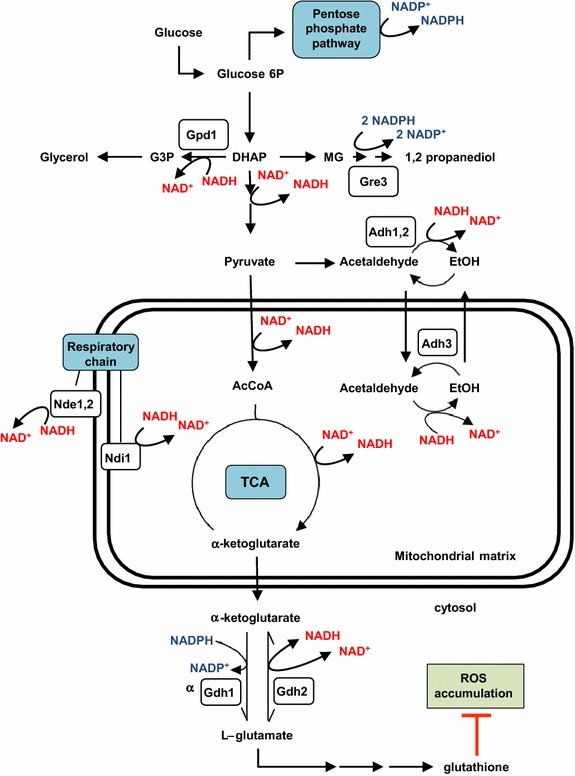
Schematic representation of some yeast NADH- and NADPH-dependent reactions and pathways cited in the text. *Adh1-3* alcohol dehydrogenase, *Gpd1* glycerol-3-phosphate dehydrogenase, *Gre3* aldose reductase, *Nde1*,*2* external NADH dehydrogenase, *Ndi1* internal NADH dehydrogenase, *AcCoA* acetyl coenzyme A, *EtOH* ethanol, *DHAP* dihydroxy acetone phosphate, *G3P* glycerol 3-phosphate, *MG* methylglyoxal. Note that the glutamate dehydrogenase isoenzyme Gdh2 usually catalyzes the catabolic reaction from l-glutamate to α-ketoglutarate. Nevertheless, when overexpressed [21] or when NH_4_^+^ is plentiful [22], contributes to the glutamate production using NADH as cofactor [19]. For other details see the *text.*

A major source of NADH mitochondrial is the synthesis of α-ketoglutarate from pyruvate [18] (Figure 1) that precedes the cytosolic NADPH-dependent production of glutamate by the activity of the glutamate dehydrogenase (GDH) isoenzymes Gdh1 and Gdh3 [19]. *GDH1* is highly expressed in actively growing cells while the Ghdh3-encoding gene shows a stationary-phase specific expression [20]. A third enzyme, Gdh2, which usually catalyzes the catabolic reaction, contributes when overexpressed [21] or when NH_4_^+^ is plentiful [22], to the glutamate production using NADH as cofactor [19] (Figure 1). Recently, a study of the yeast proteome variation following a cold-shock allowed us identifying Gdh1 among the proteins showing increased abundance at low temperature (unpublished results). By using a systems biology approach, Paget et al. [6] have also identified *GDH2* as a candidate cold growth-favoring gene. Altogether, these results suggest that regulation of the GDH activity could represent a mechanism of adaptation to low temperature, although no experimental evidences of this function have been provided.

## Results and discussion

### A high-copy number of *GDH1* or *GDH2* have distinct effects on yeast cold growth

**Figure 2:**
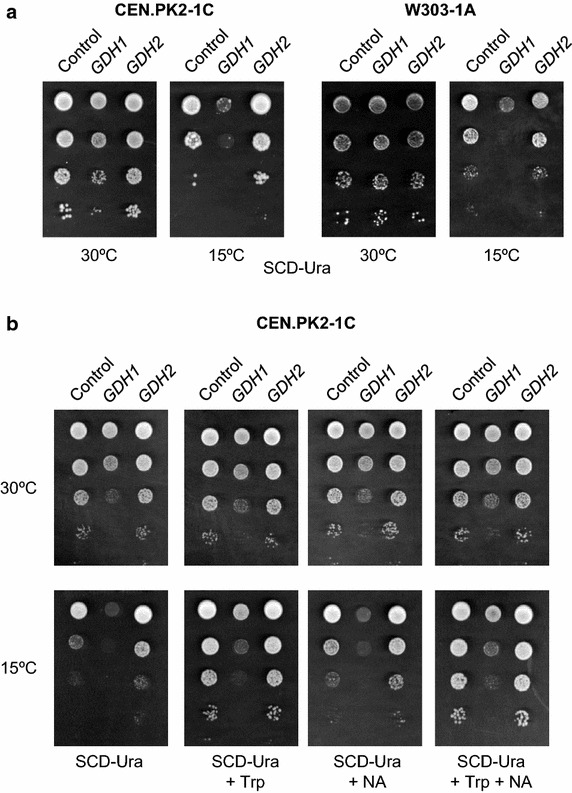
Ectopic expression of *GDH1* and *GDH2* affects the growth of *S. cerevisiae* strains at low temperature. **a** YEplac195 (*URA3*; Control, empty plasmid), YEpGDH1 (*GDH1*) and YEpGDH2 (*GDH2*) transformants of the CEN.PK2-1C and W303-1A wild-type strains were assayed for growth at low temperature. Cultures were incubated on SCD-Ura at 30°C until the exponential phase and were adjusted to OD_600_ = 1.0. Then, serial dilutions (1–10^−3^) of the cultures were spotted (3 μl) onto SCD-Ura agar medium and incubated at 30°C for 2 days or at 15°C for 10 days. **b** Growth of the CEN.PK2-1C transformants was examined on SCD-Ura medium supplemented with 200 μM of tryptophan (+Trp), 32 μM of nicotinic acid (+NA) or the same amount of both (+Trp +NA). In all cases, a representative experiment is shown.

We investigated the effects of increased glutamate dehydrogenase activity in the cold growth of *S. cerevisiae*. Yeast cells of the wild-type laboratory strain CEN.PK2-1C were transformed with plasmids YEpGDH1 and YEpGDH2, and transformants were tested for growth on synthetic SCD-Ura medium at 30 and 15°C. As it is shown in Figure 2a, overexpression of *GDH1* was detrimental for yeast growth, in particular at 15°C where YEpGDH1 transformants showed a dramatic cold-sensitive phenotype. On the contrary, increased expression of *GDH2* caused no apparent effect at 30°C and conferred a growth advantage upon cold exposure. Similar results were obtained for transformants of the W303-1A wild-type strain ruling out a strain-dependent effect (Figure 2a).

### Exogenous addition of NAD^+^ precursors stimulate yeast cold growth

Then, we examined whether the observed effects by ectopic expression of *GDH1* or *GDH2* were mediated by increased production of glutamate from α-ketoglutarate. The GDH activity plays a central role in the ammonia assimilation [23] and thus, the engineering of this metabolic reaction may have a great impact in the amino acids pool, including tryptophan. As mentioned, tryptophan metabolism has a strong influence in the ability of yeast cells to face with cold stress [3, 8]. As it is shown in the Additional file 1: Figure S1, increased availability of glutamate or aspartate in the culture medium did not affect the growth of the strains tested at either 30 or 15°C. On the contrary, increased availability of tryptophan in the culture medium (200 μM) stimulated growth at low temperature (Figure 2b). Under these conditions, the effect of a high-copy number of *GDH2* was negligible. However, cells overexpressing *GDH1* still displayed a cold-sensitive phenotype.

Like tryptophan, the addition of extra amounts of nicotinic acid (niacin) to the culture medium (4.0 mg/l), one of the NAD^+^ precursors via the salvage pathway [5, 7], provided increasing cold growth to yeast cells, but again this was unable to restore a wild-type growth to YEpGDH1 cells (Figure 2b). Neither the combined addition of extra tryptophan and nicotinic acid was able to overcome the growth defect caused by the ectopic expression of *GDH1*, although the growth of all the strains at 15°C was further improved (Figure 2b). These results suggest that the total cellular NAD levels are a limiting factor for growth at low temperature in *S. cerevisiae*, an observation that fits well with the apparent requirement of increased transcription of NAD-related genes found by thermodynamic-based analysis in cold-shocked cells [6]. Increased NAD^+^ synthesis has been also found in the cryotolerant species *Saccharomyces kudriavzevii* when its metabolome at 12°C was compared with that of a cold-sensitive wine making strain of *S. cerevisiae* [24]. Our results also suggest that the role of tryptophan as NAD^+^ precursor may account for the need of high levels of tryptophan at low temperature.

### Overexpression of *GDH1* or *GDH2* decreases ROS levels

**Figure 3:**
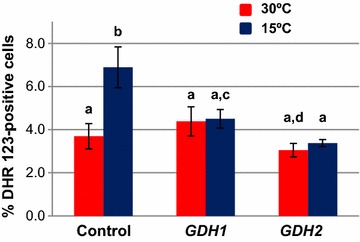
Increased activity of the glutamate dehydrogenase (GDH) isoenzymes Gdh1 and Gdh2 reduces the accumulation of ROS at low temperature. Cells of the CEN.PK2-1C strain harboring plasmids, YEplac195 (Control, empty plasmid), YEpGDH1 (*GDH1*) and YEpGDH2 (*GDH2*) were cultivated on SCD-Ura at 30°C or 15°C and tested for ROS accumulation by labeling of cells with the fluorescent probe dihydrorhodamine 123 (DHR 123). Data are expressed as the percentage of viable cells showing DHR 123-positive staining by flow cytometry. Values represent the means of at least four independent experiments. The error associated with the points was calculated by using the formula: (1.96 × SD)/√n, where SD is the standard deviation and n is the number of measurements. Values with *different letter* are significantly different with a *p* < 0.05.

l-glutamic acid is one of the precursors for glutathione biosynthesis (Figure 1), a metabolic pathway that operate through the sequential action of *GSH1*-encoded γ-glutamyl cysteine synthase and *GSH2*-encoded glutathione synthase [25]. The glutathione (GSH) pathway drives an array of cellular functions involving reversible disulfide formation and as such plays a fundamental role as redox buffer [26]. Hence, we were interested to examine whether the overexpression of *GDH1* and *GDH2* may influence the oxidative response of yeast cells. It is worth to point out that exposure of *S. cerevisiae* to suboptimal temperatures have been reported to increase the intracellular levels of H_2_O_2_ and upregulate the expression of antioxidant genes, among them *GSH1* [9]. A relationship between the NADP^+^-dependent glutamate dehydrogenase activity encoded by *GDH3* and oxidative-stress-induced apoptosis has been also established [20]. As can be seen in Figure 3, ectopic expression of either *GDH1* or *GDH2* had no effect on the percentage of 30°C-grown wild-type yeast cells labelled with dihydrorhodamine 123, a molecular fluorogenic probe for testing the accumulation of reactive oxygen species (ROS) [27]. Cold exposure caused an increase of around twofold in the percentage of wild-type cells labelled with the fluorescent ROS indicator, in consonance with previous reports [9]. However, ectopic expression of *GDH1* and *GDH2* appeared to compensate this effect and the number of dihydrorhodamine 123-positive cells did not vary significantly upon cold stress (Figure 3). Thus, the results are consistent with a role of both *GDH1* and *GDH2* in stimulating the synthesis of glutamate and preventing cold-induced ROS accumulation. Nevertheless, this activity does not appear to account for the distinct cold-growth phenotypes displayed by YEpGDH1 and YEpGDH2 cells (Figure 2). Neither, the oxidative stress induced by cold exposure seems to have a major effect on growth of yeast cells at low temperature.

### Knock-out of *GRE3* rescues the cold-growth defect of YEpGDH1 transformants

**Figure 4:**
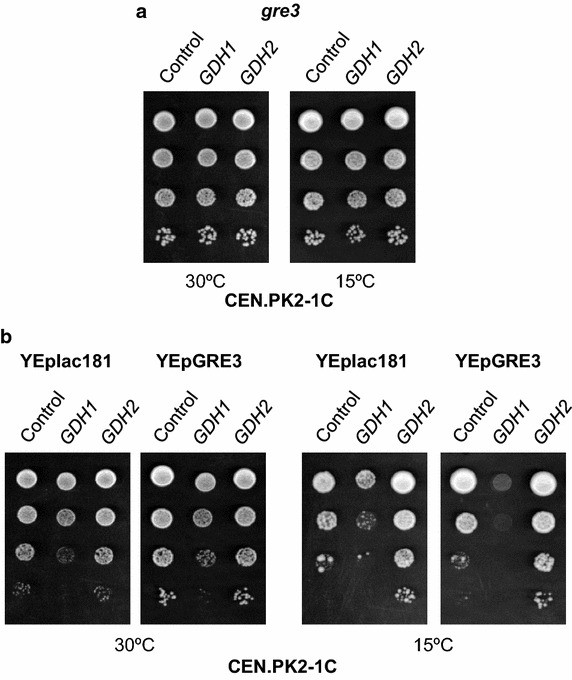
NADPH availability affects yeast cold growth. **a** CEN.PK2-1C derivatives of the *gre3* mutant strain carrying plasmids YEplac195 (*URA3*; Control, empty plasmid), YEpGDH1 (*GDH1*) and YEpGDH2 (*GDH2*) were examined for growth at 30 and 15°C on SCD-Ura agar medium. **b** CEN.PK2-1C wild-type cells carrying plasmids YEplac181 (*LEU2*; empty plasmid) or YEpGRE3, which contains the *S. cerevisiae* *GRE3* gene, and YEplac195 (*URA3*; Control, empty plasmid), YEpGDH1 (*GDH1*) or YEpGDH2 (*GDH2*) were pre-grown, diluted and spotted for growth assay at 30 and 15°C as described in the Figure 2. In all cases, a representative experiment is shown.

NAD(H) and NADP(H) play different cellular functions and are connected to specific branches of the metabolic network [5]. Thus, it is conceivable that the specific effects on cold growth observed in cells overproducing Gdh1 or Gdh2 were the consequence of the use of different cofactors for the conversion of α-ketoglutarate to glutamate (Figure 1). We first check whether increasing NADPH availability, by knock-out of the *GRE3* gene, could influence the growth of YEpGDH1 transformants. The yeast aldose reductase encoded by *GRE3* [28], transforms methylglyoxal, an intrinsic intermediate of the glycolysis [29, 30], into 1-2 propanediol in a two-step reaction dependent on NADPH [31] (Figure 1). Lack of Gre3 function stimulated the cold growth of the CEN.PK2-1C wild-type strain (Figure 4a; compare with control cells, Figure 2a, 15°C). Hence, increased availability of NADPH appears to favour yeast cold-growth, suggesting a limited supply/production of the cofactor under these conditions. Furthermore, the growth defects produced by ectopic expression of *GDH1* were completely eliminated by knock-out of *GRE3* (Figure 4a). Finally, the combined overexpression of *GDH1* and *GRE3* produced the most severe cold-sensitive phenotype among the strains analysed (Figure 4b). Thus, our results suggest that the cold-growth defect observed in YEpGDH1 transformants is caused by an excess of NADPH consumption.

### The oxidation of NADH to NAD^+^ is critical for cold growth

**Figure 5:**
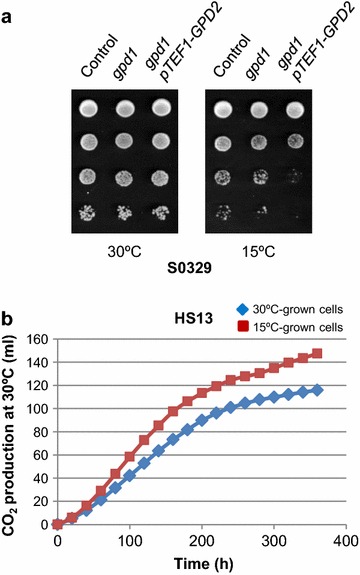
Exposure to low temperature increases the fermentative/oxidative balance and the need for glycerol synthesis. **a** Growth of the S0329-derivative yeast strains, wild-type (Control), *gpd1* mutant (*gpd1*), and *gpd1* containing in addition a replacement of the native promoter of *GPD2* by a mutated promoter of the unregulated *TEF1* yeast gene (*gpd1* *pTEF1*-*GPD2*) was examined as described in the Figure 2. **b** Time–course graph of total gas produced for 360 min by cell suspensions of the baker’s yeast strain HS13 grown at 30 or 15°C. Cells were collected and the amount of CO_2_ evolved at 30°C recorded in a Fermograph II apparatus as described in the “Methods” section. A representative experiment is shown.

Then, we examined whether changes in the NADH/NAD^+^ ratio might also impact the growth behaviour of yeast cells at different temperatures and account for the cold-tolerant phenotype of YEpGDH2 cells. In *S. cerevisiae* the reduction of NAD^+^ to NADH is coupled to the conversion of glucose to pyruvate by the glycolytic pathway (Figure 1). Additionally, mitochondrial NADH is generated by the tricarboxylic acid (TCA) cycle or by biosynthetic reactions. Under full respiratory conditions, the oxidation of NADH is mainly coupled to the mitochondrial energy generation. On the contrary, when the capacity of the respiratory pathways is limited the oxidation of cytosolic NADH lies on the formation of ethanol, and glycerol [32], the main yeast redox sink [33, 34]. In parallel, the ethanol-acetaldehyde shuttle, which involves the mitochondrial alcohol dehydrogenase Adh3 [35] (Figure 1), plays a key role in the maintenance of the mitochondrial redox balance [36]. Quite remarkably, cells of *S. cerevisiae* lacking the *ADH3* gene has been reported to show decreased fitness at low temperature, while its overexpression was found to stimulate cold growth [6]. Studies have also shown that cold exposure increases glycerol production [37] and that the ability of certain species of *Saccharomyces* as *S. kudriavzevii* to grow at low temperature correlates with enhanced enzymatic activity of glycerol-3-phosphate dehydrogenase [38], the enzyme encoded by *GPD1* [39], and its homologue *GPD2* [33]. Consistent with this, we found that decreased capacity of glycerol production reduces cold growth in *S. cerevisiae*. As can be seen in Figure 5a, knock-out of *GPD1* in cells of the yeast wild-type strain S0329 had no major effect on growth at low temperature. However, *gpd1*Δ cells in which the glycerol production was further reduced by replacement of the native *pGPD2* by a weaker mutated constitutive *pTEF1* promoter [40] displayed a strong cold-sensitive phenotype (Figure 5a). These results suggest that exposure to low temperature compromises the cellular capacity to keep a proper NADH/NAD^+^ balance and that increasing NADH oxidation by overexpression of *GDH2* helps to avoid perturbations in the redox metabolism.

### Cold exposure endows yeast cells with higher fermentative capacity

In *S. cerevisiae*, a limitation in respiratory capacity results in “overflow metabolism” leading to NADH accumulation and the formation of ethanol and glycerol, a phenomena that can be reduced, among others, by overexpression of heterologous NADH oxidases [41]. Thus, the most obvious explanation for the results showed above is that cold exposure induces an increase in the fermentative/oxidative balance most likely as a consequence of a temperature dependency of both oxygen solubility and respiration rate [15]. Consistent with this, previous studies have reported an increase of fermentative capacity in yeast cells grown in anaerobic chemostat conditions at low temperature [16, 42], a result that have not been explored in air-exposed cultures of baker’s yeast strains.

With this idea, cells of the industrial baker’s yeast strain HS13 were grown on molasses plates at 30 or 15°C and their fermentative capability was analyzed in liquid dough preparations. As shown in Figure 5b, yeast biomass propagated at 15°C showed an increased CO_2_ production kinetics (recorded at 30°C) as compared with that of 30°C-grown cells. For instance, CO_2_ production attained by 15°C-grown cells for 180 min (105.0 ± 2.7 ml CO_2_), was about 20% higher than that observed with 30°C-grown cells (87.1 ± 3.3 ml CO_2_; *p* < 0.05). Hence, a reduction in ambient temperature induces a shift toward increased fermentative metabolism in *S. cerevisiae*.

### Increasing *GDH2* activity stimulates cold growth of the QA23 wine yeast strain

**Figure 6:**
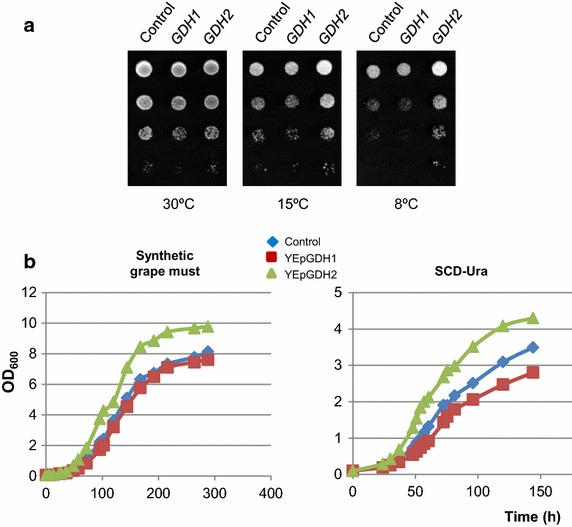
Overexpression of *GDH2* improves the cold performance of the QA23 *ho* wine yeast strain. **a** Ura^−^ derivatives of the wine strain QA23 *ho* were transformed with YEplac195-based plasmids (*URA3*) containing *GDH1* or *GDH2* and assayed for growth at the indicated temperatures on SCD-Ura agar medium. Transformants carrying the empty plasmid were also tested (Control). In all cases, cells were pre-grown and treated as described in the Figure 2. **b** The same strains were tested for growth at 12°C in synthetic grape must (*left graph*) or liquid SCD-Ura (*right graph*) medium. Growth of yeast cultures was monitored by measuring the cell suspension’s optical density at 600 nm (OD_600_) for the indicated period. A representative experiment is shown.

High growth at low temperature has become one of the most important oenological criteria used to select industrial wine yeast strains. Uncovering genes and metabolic pathways that determine the adaptation of industrial yeast strains to cold is therefore highly relevant to targeting the genetic improvement of these microorganisms. Therefore, we investigated whether the ectopic expression of *GDH1* and *GDH2* in a wine yeast strain may have a similar impact on cold growth as that observed in lab yeast strains. Industrial wine strains differ genetically and physiologically from *S. cerevisiae* lab strains [43] and thus changes in the copy number of a particular gene might result in distinct responses. To address this, an Ura^−^ auxotrophic derivative (MJHL201, *ura3*) of the QA23 *ho* wine strain [44] was transformed with the *URA3*-based plasmids YEpGDH1 and YEpGDH2 and tested for growth at low temperature in SCD-Ura plates. Enhanced growth was observed for YEpGDH2 transformants exposed to a range of low temperatures from 8 to 15°C (Figure 6a). Overexpression of *GDH1* impaired again cold-growth although its effects appeared less pronounced in the prototroph wine yeast (Figure 6a) than those found in laboratory strains (Figure 2). Similar results were observed in SCD-Ura liquid cultures at 12°C (Figure 6b, right graph). In this point, we investigated the cold response of the wine yeast transformants in a synthetic grape must. As it is shown, the overexpression of *GDH1* had no noticeable effects on cold growth (Figure 6b, left graph), a result that stress the importance of checking physiological responses in wine yeasts under these conditions. However, ectopic expression of *GDH2* stimulated again the growth at low temperature of the QA23 strain (Figure 6b, left graph), confirming thus the data for laboratory strains of *S. cerevisiae* and the potential of this tool to manipulate the redox balance and the performance of industrial strains at low temperature.

## Conclusions

Exposure of yeast cells to temperatures below the optimal increases the fermentative/oxidative balance. In this scenario, the oxidation of NADH is a fundamental requirement to avoid overflow metabolism and allow sustained growth. This accounts for the distinct effects on cold growth display by *GDH1* or *GDH2* redox-engineered strains of *S. cerevisiae*. Increased activity of the NADPH-dependent glutamate dehydrogenase encoded by *GDH1* renders cells cold-sensitive, most likely due to NADPH depletion, whereas the Gdh2-dependent NAD^+^ supply stimulates the growth at low temperature. This provides new insights about the adjustment of the physiological state of yeast cells in response to variations of ambient temperature and identifies redox factors that limit or promote cold fermentation processes. Whether increased NADH oxidation by overexpression of *GDH2* alters the distribution of metabolic fluxes in the cell and the production of by-products, as it has been reported for strains expressing heterologous NADH oxidases [41, 45], needs to be clarified in future studies. Quite remarkably, our data point out an important role of elevated Gdh2 activity in promoting cold growth. Adaptive evolution approaches targeted to this redox reaction might be successful for the selection and improvement of industrial strains, avoiding the consumer’s reticence with respect to genetically modified organisms.

## Methods

### Media and culture conditions

Yeast cells were cultured at 30°C in defined media, YPD (1% yeast extract, 2% peptone, 2% glucose) or SCD [0.17% yeast nitrogen base without amino acids (DIFCO), 0.5% ammonium sulfate, 2% glucose] supplemented with the appropriate drop-out mixtures (ForMedium, England). Synthetic grape must was prepared as described by Riou et al. [46]. For gas production assays, YPD-grown cells were inoculated onto 140 mm-diameter plates (10 units of OD_600_ per plate) containing molasses agar [5.0 g l^−1^ of beet molasses (49% sucrose), 0.5 g l^−1^ of (NH_4_)_2_HPO_4_, 26.0 g l^−1^ of agar, and 20 mg l^−1^ of biotin; adjusted to a final pH of 5.0]. Plates were incubated for 22 or 48 h at 30 and 15°C, respectively. Yeast transformants carrying the geneticin (kanMX4) resistant module were selected on YPD agar plates containing 200 mg/l of G-418 (Sigma) [47]. *Escherichia coli* DH5α host strain was grown in Luria–Bertani (LB) medium (1% peptone, 0.5% yeast extract and 0.5% NaCl) supplemented with ampicillin (50 mg/l). All amino acids, sugars and antibiotics were filter-sterilized and added to autoclaved medium. Solid media contained 2% agar. Yeast cells were transformed by the lithium acetate method [48]. *E. coli* was transformed by electroporation following the manufacturer’s instructions (Eppendorf). Plate phenotype experiments were made by diluting the cultures to OD_600_ = 1.0 and spotting (3 μl) tenfold serial dilutions. Unless indicated, colony growth was inspected after 2 and 10–12 days of incubation at 30 and 15°C, respectively.

### Strains and plasmids

*Saccharomyces cerevisiae* CEN.PK2-1C (*MATa ura3*-*52 his3*-∆*1 leu2*-*3*,*112 trp1*-*289 MAL 2*-*8*^*c*^*SUC2*) [49] and W303-1A (*MATaade2*-*1 his3*-*11*,*15 leu*-*2*-*3*,*112 trp1*-*1 ura3*-*1 can1*-*100 GAL mal SUC2*) [50] wild type strains were used throughout this work. Strains S0329 (*MATa trp1*-*1 leu2*-*3*,*112 ura3*-*52 his4 can1 FUS1*::*lacZ*::*LEU2*) [51] and PW401 (S0329 *gpd1*::*URA3*) [52] were a gift of J. Thorner. The baker’s yeast strain HS13 (Lesaffre International, Lille, France) was also used in some experiments. Construction of the QA23 *houra3* mutant strain (MJH201), an Ura^−^ haploid derivative of the wine yeast strain QA23 (Lallemand, Montreal, Canada), was previously described [44]. PCR-amplified fragments containing the whole sequence of *GDH1* and *GDH2* gene, including its own promoter and terminator were obtained with specific synthetic oligonucleotides (Additional file 2: Table S1) and genomic DNA as template. Amplifications were carried out under standard conditions. The corresponding fragments were cloned into the pGEM^®^-T vector (Promega) obtaining the plasmids pGEM-GDH1 and pGEM-GDH2. Then, EcoRI/SSpI (pGEM-GDH1) and BglII/PvuII (pGEM-GDH2) fragments were released and ligated into the plasmid YEplac195 (*URA3*, [53]) previously digested with EcoRI/SmaI and BamHI/SmaI, respectively, resulting in plasmids YEpGDH1 and YEpGDH2. Plasmid p416-TEFmut4-yECitrine which allows the PCR-based construction of a replacement cassette of the native *GPD2* promoter by a mutated *TEF1* promoter [40] was kindly provided by E. Nevoigt. The YEpGRE3 plasmid, a derivative of the YEplac181 (*LEU2*, [53]) shuttle vector, was constructed previously [54]. The deletion cassette for *GRE3* was prepared by PCR using specific synthetic oligonucleotides (Additional file 2: Table S1) and plasmid pFa6A (kanMX4) as template, respectively [47]. Detection of the correct gene disruption or promoter replacement was done by diagnostic PCR [55], using a set of oligonucleotides (Additional file 2: Table S1), designed to bind outside of the replaced gene sequence and within the marker module (data not shown).

### Measurement of intracellular ROS by flow cytometry

Five milliliters of SCD-Ura medium were inoculated at OD_600_ = 0.25 from overnight seed cultures of YEplac195, YEpGDH1 and YEpGDH2 transformants of the CEN.PK2-1C strain, and cultivated at 30°C. When OD_600_ reached 0.5, aliquots were withdrawn for their immediate analysis (30°C-control), and cultures were transferred to 15°C for 18 h (15°C-samples). Cells were harvested by centrifugation, washed twice with 10 mM PBS (pH 7.2) and resuspended in the same buffer (0.25 units of OD_600_ per ml). Then, dihydrorhodamine 123 (DHR 123, Sigma) was added at 5 μg per ml of cell culture from a 2.5 mg/ml stock solution in ethanol and cells were incubated in the dark for 90 min at 28°C. Finally, cells were harvested, washed, resuspended in PBS buffer and analysed using the “Annexin V and Cell Death” channel of a flow cytometer Muse Cell Analyzer (Millipore). The settings were adjusted using negative (DHR 123-untreated cells) and positive (4 mM H_2_O_2_/60 min-stressed cells treated with DHR 123) controls. Data are expressed as the percentage of cells showing DHR 123-positive staining. The data represent the mean ± SE of four independent experiments.

### CO_2_ production assays

Molasses-grown cells (30 or 15°C) were collected, washed, resuspended and the final yeast concentration adjusted to 30 mg (dry weight) per ml as previously described [56]. Fifteen milliliters of the yeast mixture was poured into a 100-ml screw cap graduated bottle, placed in a 30°C water bath and gently shaken (80 rpm). After 15 min, 15 ml of 30°C-pre-warmed 2× liquid dough model solution [57] was added and the amount of CO_2_ evolved recorded in a Fermograph II apparatus (ATTO Co., Ltd., Tokyo, Japan). Values are expressed as ml of CO_2_ and represent the mean ± SE of total volume of gas produced after 180 min of fermentation or are shown as the time–course graph of total gas. At least four independent experiments were conducted for each yeast strain.

### Statistical analysis

Sample averages were compared using a Student’s *t* test. The samples denoted with different letter were significantly different with a *p* < 0.05.

## Additional files

Additional file 1:
**Figure S1.** Increased availability of aspartate or glutamate does not alter the cold growth phenotype provided by ectopic expression of *GDH1* and *GDH2*. YEplac195 (*URA3*; Control, empty plasmid), YEpGDH1 (*GDH1*) and YEpGDH2 (*GDH2*) transformants of the CEN.PK2-1C wild-type strain were assayed for growth at low temperature. Cultures were incubated on SCD-Ura at 30°C until the exponential phase and were adjusted to OD_600_=1.0. Then, serial dilutions (1–10^−3^) of the cultures were spotted (3 μl) onto SCD-Ura agar medium supplemented with 800 μg/ml of aspartate (SCD-Ura + Asp) or glutamate (SCD-Ura + Glu) and incubated at 30°C for 2 days or at 15°C for 10 days. A representative experiment is shown. Additional file 2:
**Table S1.** Oligonucleotides used in this study.
