# Supplementary material for: Redox engineering by ectopic expression of glutamate dehydrogenase genes links NADPH availability and NADH oxidation with cold growth in Saccharomyces cerevisiae
Source: Microb Cell Fact. 2015 Jul 9;14:100. doi: 10.1186/s12934-015-0289-2 (PMC4496827; doi:10.1186/s12934-015-0289-2)
Supplement: Additional file 1: — Figure S1. Increased availability of aspartate or glutamate does not alter the cold growth phenotype provided by ectopic expression of GDH1 and GDH2. YEplac195 (URA3; Control, empty plasmid), YEpGDH1 (GDH1) and YEpGDH2 (GDH2) transformants of the CEN.PK2-1C wild-type strain were assayed for growth at low temperature. Cultures were incubated on SCD-Ura at 30°C until the exponential phase and were adjusted to OD600=1.0. Then, serial dilutions (1–10−3) of the cultures were spotted (3 μl) onto SCD-Ura agar medium supplemented with 800 μg/ml of aspartate (SCD-Ura + Asp) or glutamate (SCD-Ura + Glu) and incubated at 30°C for 2 days or at 15°C for 10 days. A representative experiment is shown. [file 12934_2015_289_MOESM1_ESM.pptx]

## Slide 1
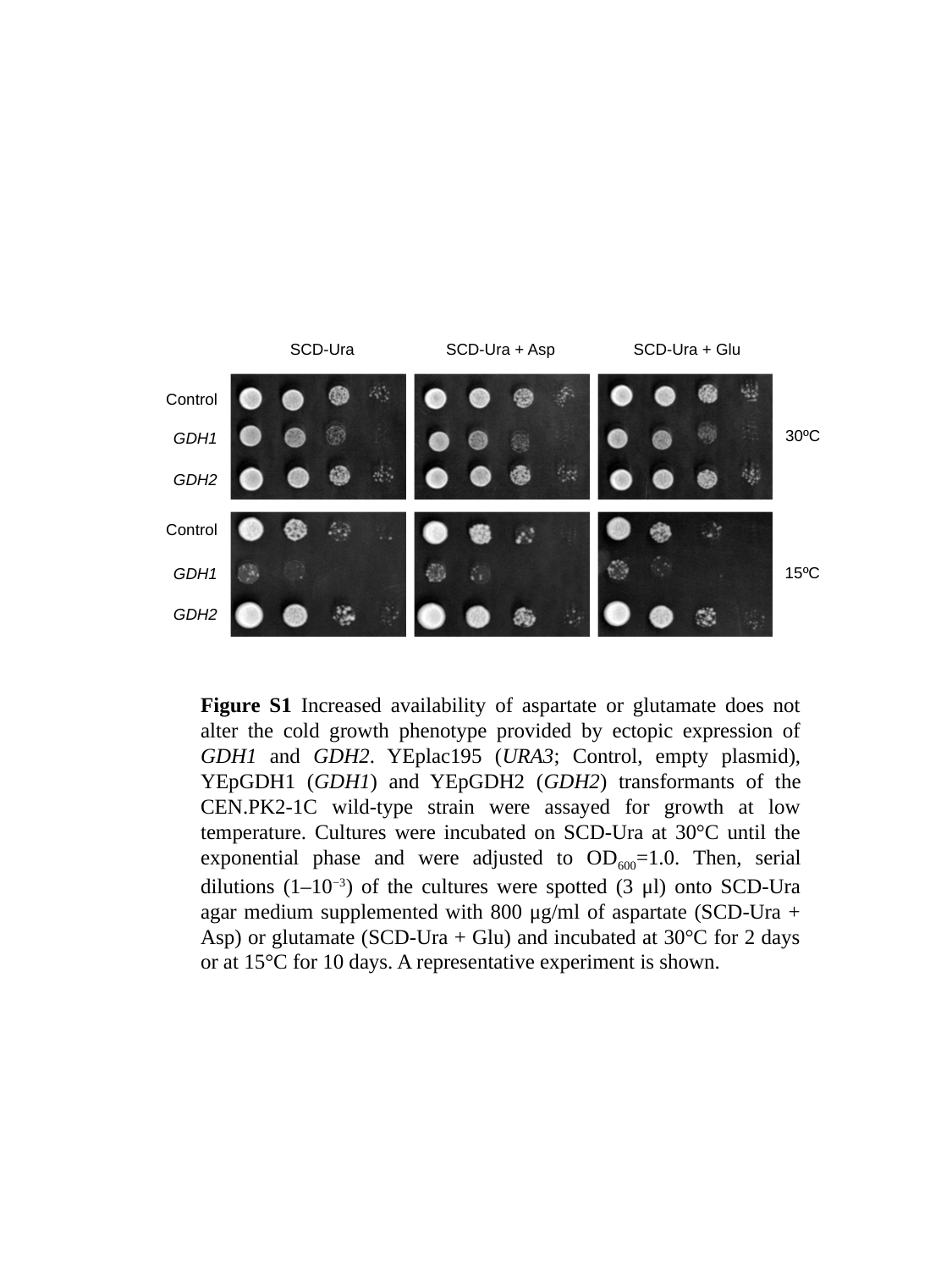

SCD-Ura
SCD-Ura + Asp
SCD-Ura + Glu
Control
30ºC
GDH1
GDH2
Control
15ºC
GDH1
GDH2
Figure S1 Increased availability of aspartate or glutamate does not alter the cold growth phenotype provided by ectopic expression of GDH1 and GDH2. YEplac195 (URA3; Control, empty plasmid), YEpGDH1 (GDH1) and YEpGDH2 (GDH2) transformants of the CEN.PK2-1C wild-type strain were assayed for growth at low temperature. Cultures were incubated on SCD-Ura at 30°C until the exponential phase and were adjusted to OD600=1.0. Then, serial dilutions (1–10−3) of the cultures were spotted (3 μl) onto SCD-Ura agar medium supplemented with 800 μg/ml of aspartate (SCD-Ura + Asp) or glutamate (SCD-Ura + Glu) and incubated at 30°C for 2 days or at 15°C for 10 days. A representative experiment is shown.
