# Supplementary material for: Redox engineering by ectopic expression of glutamate dehydrogenase genes links NADPH availability and NADH oxidation with cold growth in Saccharomyces cerevisiae
Source: Microb Cell Fact. 2015 Jul 9;14:100. doi: 10.1186/s12934-015-0289-2 (PMC4496827; doi:10.1186/s12934-015-0289-2)
Supplement: Additional file 2: — Table S1. Oligonucleotides used in this study. [file 12934_2015_289_MOESM2_ESM.docx]

**Table S1 Oligonucleotides used in this study**

| Name | Sequence | Used for |
| --- | --- | --- |
|  |  |  |
| GRE3-K1 | ATGTCTTCACTGGTTACTCTTAATAACGGTCTGAAAATGC  CCCTACGTACGCTGCAGGTCGAC | Deletion *GRE3* |
| GRE3-K2 | TCAGGCAAAAGTGGGGAATTTACCATCCAACCAGGT  CCATGGATCATCGATGAATTCGAGCTCG | Deletion *GRE3* |
| GRE3-V1 | ACTCCTGGTACACTGAAG | Verification deletion *GRE3* |
| GDH1-1 | CTTGAATTCTTCTAGCGG | PCR-amplification of *GDH1* |
| GDH1-2 | GGAAATGTCTGTTGATGC | PCR-amplification of *GDH1* |
| GDH2-1 | TATGATAACGAGAAGATTTC | PCR-amplification of *GDH2* |
| GDH2-2 | TTTGAAAGTGACATGCTG | PCR-amplification of *GDH2* |
| P7-GPD2 | TAGCTTACGGACCTATTGCCATTGTTATTCCGATTAATCTATTGT  CAGCTGAAGCTTCGTACG | *pGPD2* replacement cassette |
| P8c-GPD2 | TGCGTTCGCTTAAGGAATGTGTATCTTGTTAATCTTCTGACAGCA  AGCATTTTTCTAGAGAACTTAG | *pGPD2* replacement cassette |
| GPD2-V1 | AAGACGACGATGGCTCTG | Verification *pGPD2* replacement |
| Kan-S2 | GTCAAGGAGGGTATTC | Verification deletion *GRE3* and *pGPD2* replacement |
